# 

**Published:** 1900-06-02

**Authors:** 


### NOTICE TO CORRESPONDENTS.

All MS., Letters, Books for Review, and other matters intended for the Editor should be addressed The Editor, "The Hospital," The Hospital Building, 28 & 29, Southampton Street, Strand, W.C.

The Editor cannot undertake to return rejected MS., even when accompanied by stamped directed envelope.

**Notice to Advertisers and Subscribers.**

All Advertisements, Orders for Copies of The Hospital, and Business Communications should be addressed to **THE MANAGER (Not to the Editor)**, The Hospital, The Hospital Building, 28 & 29, Southampton Street, Strand, London, W.C.

The Cost of Subscription, post free, is as follows:—Per week, 2½d.; per quarter, 2s. 8d.; per half-year, 5s. 3d.; per annum, 10s. 6d. Foreign:—Per annum, 15s. 2d., payable, in all cases, in advance.

**NOTICE.—Vol. XXVII. of "The Hospital" (October, 1899, to April, 1900), in handsome cloth binding, is now on sale at the Office of this Journal, price 7s. 6d. Cases for binding the Numbers contained in this Volume 1s. 6d. Index to Vol. XXVII., 2½d., post free.**

**The Monthly Part for June is now ready. Price 6d., by post 8d.**

### NOTES ON BOOKS.

The album which has been designed as a souvenir and official programme of the National Bazaar at the Empress Rooms is a sumptuous volume full of good stuff. The bazaar being in aid of the sufferers by the war in South Africa, the album also naturally deals mostly with warlike subjects, and more especially with the various regiments represented by stalls in the bazaar. The literary and artistic pages contain contributions on many matters from a long list of well-known people, and these, with the illustrations which are profusely scattered throughout its pages, will certainly render the album of much more than mere ephemeral interest. It forms a very handsome and most interesting souvenir, and the greatest credit is due to its publishers, Messrs. Langfier, and Messrs. Gale and Polden, for the admirable manner in which it has been produced in the short space of three weeks.

Among the examples of mingled business and patriotism which we meet with at every turn we would draw attention to an edition of Rudyard Kipling's Absent-Minded Beggar, issued by the Printing Arts Company. It is an admirable example of colour printing, and although it is frankly stated that the object of the publication is to introduce this style of printing to the English public it is arranged that each copy sold shall bring threepence to the Absent-Minded Beggar Relief Fund organised by the *Daily Mail*.

### BOOKS RECEIVED.

Oliver and Boyd.
"The Physical Basis of Memory." By William Elder, M.D., F.R.C.P.

A. Malone, Paris.

"Manuel Complet de Gynecologie Medicale et Chirurgicale." Par A. Lutaud. Nouvelle edition.

Strand Newspaper Company.
"Passmore Edwards Institutions." By J. J. Macdonald.

Isbister and Co.
"The Magic Word." By Constance Smith.
"A Brave Poor Thing." By L. T. Meade.

### THE STUDENT OF MEDICINE.

### APPOINTMENTS.

R. T. BAILEY, M.R.C.S.Eng., L.R.C.P.Lond., appointed House Physician to the Liverpool Royal Infirmary. J. Blamey, M.R.C.S.Eng., appointed Medical Officer for the Penrhyn District of the Falmouth Union. J. M. Campbell, M.B., C.M.Glasg., appointed Certifying Factory Surgeon for Pollokshaws District, co. Renfrew. B. R. C. Christie, M.B., C.M.Edin., appointed Resident House Surgeon to the Glasgow Royal Infirmary. C. R. Dykes, M.R.C.S.Eng., L.R.C.P.Lond., appointed Medical Officer for the Evercreech District of the Shepton Mallet Union. J. H. Harris, M.R.C.S.Eng., D.P.H.Camb., appointed Medical Officer of Health to the Dartmouth and Totnes Port Sanitary Authority. D. Hennessy, L.R.C.P., L.R.C.S.Edin., L.F.P.S.Glasg., appointed Certifying Factory Surgeon for the Bandon, Kilbrogan, and Brinny Electoral Divisions, co. Cork. C. G. Higginson, M.R.C.S., L.R.C.P., L.S.A., M.A.Lond., appointed Assistant Medical Officer to the Birmingham Infirmary. J. J. Kinsella, M.B., B.Ch., B.A.O.R.U.I.. appointed Certifying Factory Surgeon for the Edenderry Poor-law Union, King's County. W. Kirkby, L.R.C.P., L.R.C.S.Edin., appointed Medical Officer of Health to the Maesteg Urban District Council. R. Owen, L.R.C.P., L.R.C.S.Edin., appointed Medical Officer for the Llandwrog District of the Carnarvon Union. E. F. Potter, M.D., appointed to the Senior Staff of the London Throat Hospital. J. S. Rosser, M.D.Glasg., D.P.H., F.P.S.Glasg., appointed Certifying Factory Surgeon for the Llandovery District. K. Scott, F.R.C.S., appointed Ophthalmic Surgeon to the St. Mary's Children's Hospital, Plaistow. C. M. Vernon, M.R.C.S., L.R.C.P.Lond., appointed Medical Officer for the Workhouse of the West Ashford Union. A. S. Tindal, M.D., appointed Assistant Physician to the Victoria Infirmary, Glasgow.

### BOOKS FOR NURSES.

Domville's Manual for Hospital Nurses, 8th edition ... ... ... ... ... ... ... 2/6

Cullingworth's Monthly Nursing, 4th edition... 1/6

Hellier's Gynæcological Nursing... ... ... 1/6

Cuff's Lectures on Medicine to Nurses .. ... 3/6

Richmond's Antiseptic Principles for Nurses 1/-

Mayne's Short Dictionary of Medical Terms, 2/6

Chavasse's Advice to a Wife, 14th edition, by Dr. Fancourt Barnes ... ... ... ... 2/6

Chavasse's Advice to a Mother, 15th edition, by Dr. George Carpenter .. ... ... ... 2/6

London: J. & A. Churchill, 7, Great Marlborough Street.

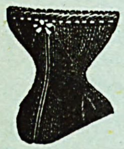

### IMPROVED KNITTED CORSETS,

**Support Without Pressure. Good and Unshrinkable.**

Improved Cotton and Pure Woollen Underclothing for Ladies, Gentlemen, and Children. Surgical Hosiery, Belts, &c. Write for Illustrated Price List and Pattern Card free. Mention Hospital. BEST SERGE KNICKERS 3/9.

KNITTED CORSET AND CLOTHING COMPANY,
118, Mansfield Road, Nottingham.

### THE NURSE'S REPORT BOOK.

Foolscap 4to., Black cover, **6d.** net. By Post 8d.

**Compiled by K. H. (Cert.)**

Containing space for general details at the beginning of the book, and for general remarks on the case at the end of the book, and with space for 200 entries of each of the following details: **Time—Nourishment—Stimulants — Temperature — Pulse — Respiration — Urine—B.O.—Sleep—Remarks.**

H. J. Glaisher, 57, Wigmore Street, Cavendish Square, W.

### NOW READY,

A REPRINT OF

### A NURSING HANDBOOK

TOGETHER WITH S. MAW, SON, AND THOMPSON'S

### COMPLETE CATALOGUE OF NURSING REQUISITES.

The above work is now republished at a cost of 1s. per copy. Messrs. S. M., S., and T. have been compelled to make this charge on account of the extreme popularity of the work and the great expense incurred in its production.

***Post Free on receipt of 1s.***

### S. MAW, SON, and THOMPSON,

7 to 12, ALDERSGATE STREET, LONDON, E.C.

